# Proceedings of the Strategy Meeting for the Development of an International Consortium for Chinese Medicine and Cancer

**DOI:** 10.1200/JGO.2016.005710

**Published:** 2016-08-31

**Authors:** Jeffrey D. White, Hongsheng Lin, Libin Jia, Roy S. Wu, Stephen Lam, Jie Li, Jinhui Dou, Nagi Kumar, Lizhu Lin, Lixing Lao

**Affiliations:** **Jeffrey D. White**, **Libin Jia**, and **Roy S. Wu**, National Cancer Institute, Rockville; **Jinhui Dou**, Food and Drug Administration, Silver Spring, MD; **Hongsheng Lin** and **Jie Li**, China Academy of Chinese Medical Sciences, Beijing; **Lizhu Lin**, First Affiliated Hospital of Guangzhou University of Chinese Medicine, Guangzhou; **Lixing Lao**, The University of Hong Kong, Pokfulam, Hong Kong, Special Administrative Region, People’s Republic of China; **Stephen Lam**, British Columbia Cancer Agency, Vancouver, British Columbia, Canada; and **Nagi Kumar**, Moffitt Cancer Center, University of South Florida, Tampa, FL.

## Abstract

On November 3, 2014, in Bethesda, MD, the Office of Cancer Complementary and Alternative Medicine of the National Cancer Institute held a meeting to examine the potential utility and feasibility of establishing an international consortium for Chinese medicine and cancer. There is significant interest in the West in using components of Chinese medicine (CM) —such as botanicals and herbal medicines, acupuncture and acupressure, and qigong—in the field of oncology, as potential anticancer agents, for symptom management, and to improve quality of life. The proposal for a consortium on CM came from the Chinese Academy of Chinese Medical Sciences, with the aims of improving scientific communications and collaborations and modernizing the studies of CM for cancer. The US National Cancer Institute’s Office of Cancer Complementary and Alternative Medicine agreed to work with Chinese Academy of Chinese Medical Sciences to explore the feasibility of establishing an international consortium for Chinese medicine and cancer. At the meeting, participants from the United States, China, Canada, Australia, and Korea discussed issues in CM and cancer research, treatment, and management, including potential mechanisms of action, proof of efficacy, adverse effects, regulatory issues, and the need for improving the quality of randomized clinical trials of CM treatments and supportive care interventions. Presented in these proceedings are some of the main issues and opportunities discussed by workshop participants.

## INTRODUCTION

Increasingly, patients and health care providers in Western countries have begun using components of Chinese medicine (CM) —such as botanicals and herbal medicines, acupuncture and acupressure, and qigong—to aid in managing various medical conditions. Simultaneously, research on the effectiveness and mechanism of action of these approaches has also expanded. In the field of oncology, many of these treatment approaches have been explored, both as potential anticancer agents and symptom management methods. Between 2005 and 2012, the research portfolio of the US National Cancer Institute (NCI) included more than 200 grants or other projects examining aspects of CM (Table 1). The research focus of these projects ranges across the broad spectrums of cancer prevention, treatment, and symptom management research. Some of these grants have studied interventions that have advanced to clinical trials in the United States, such as the oral protein-bound polysaccharide mixture derived from the mushroom *Trametes versicolor*, polysaccharide K (PSK), which is being tested as an adjuvant to an anticancer vaccine in women with metastatic breast cancer.^1^

**Table 1:**
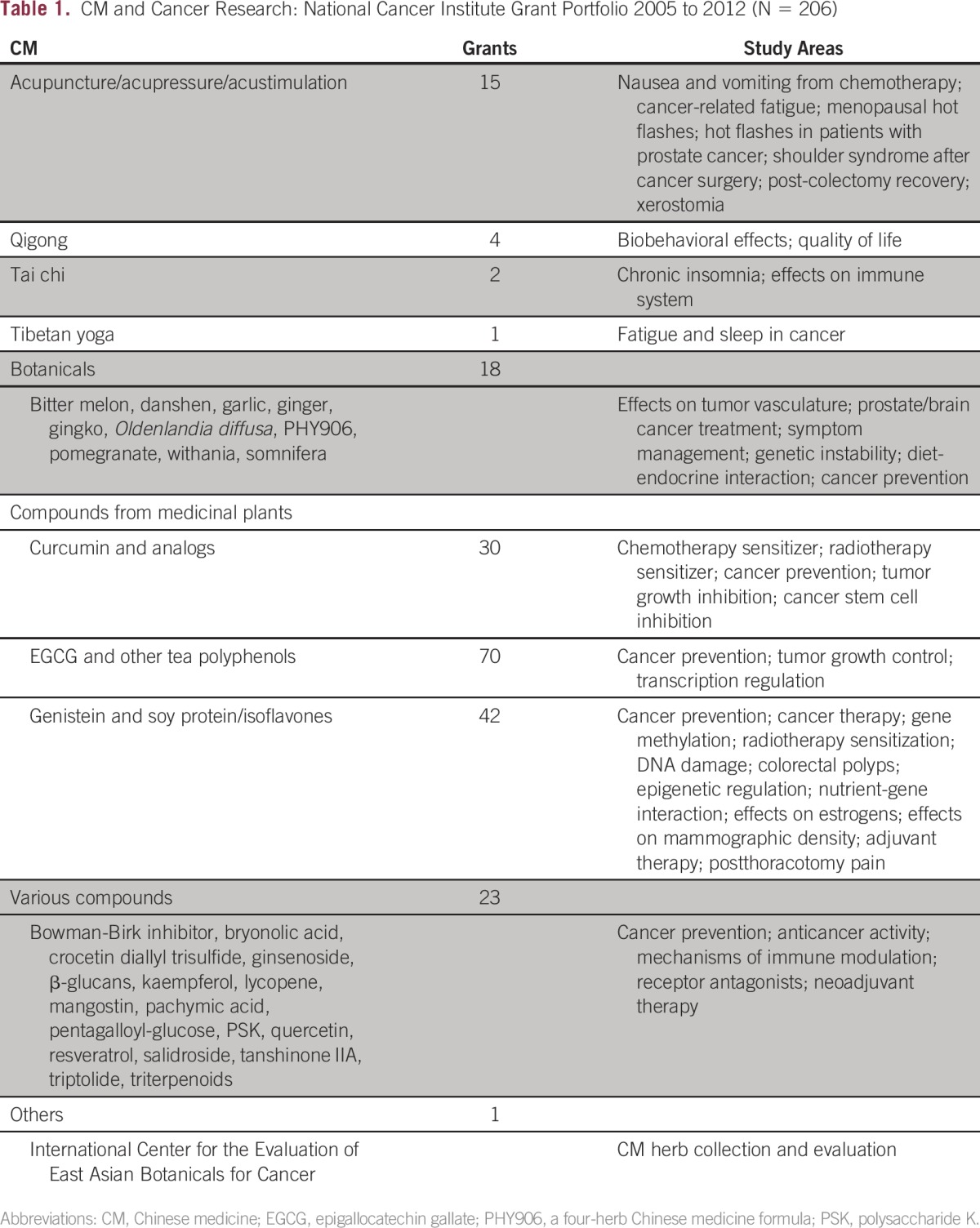
CM and Cancer Research: National Cancer Institute Grant Portfolio 2005 to 2012 (N = 206)

In April 2006, the US National Cancer Institute’s Office of Cancer Complementary and Alternative Medicine (OCCAM) hosted a conference titled “Traditional Chinese Medicine and Cancer Research: Fostering Collaborations; Advancing the Science” at the National Institutes of Health (NIH), which almost 200 scientists and physicians attended, including more than 40 from China.^2^ This meeting served as an incubator for establishing new collaborative relationships between CM and Western medicine practitioners and researchers for cancer prevention, treatment, and palliation. Since then, many new collaborations have been established (Table 2).^3-16^

**Table 2:**
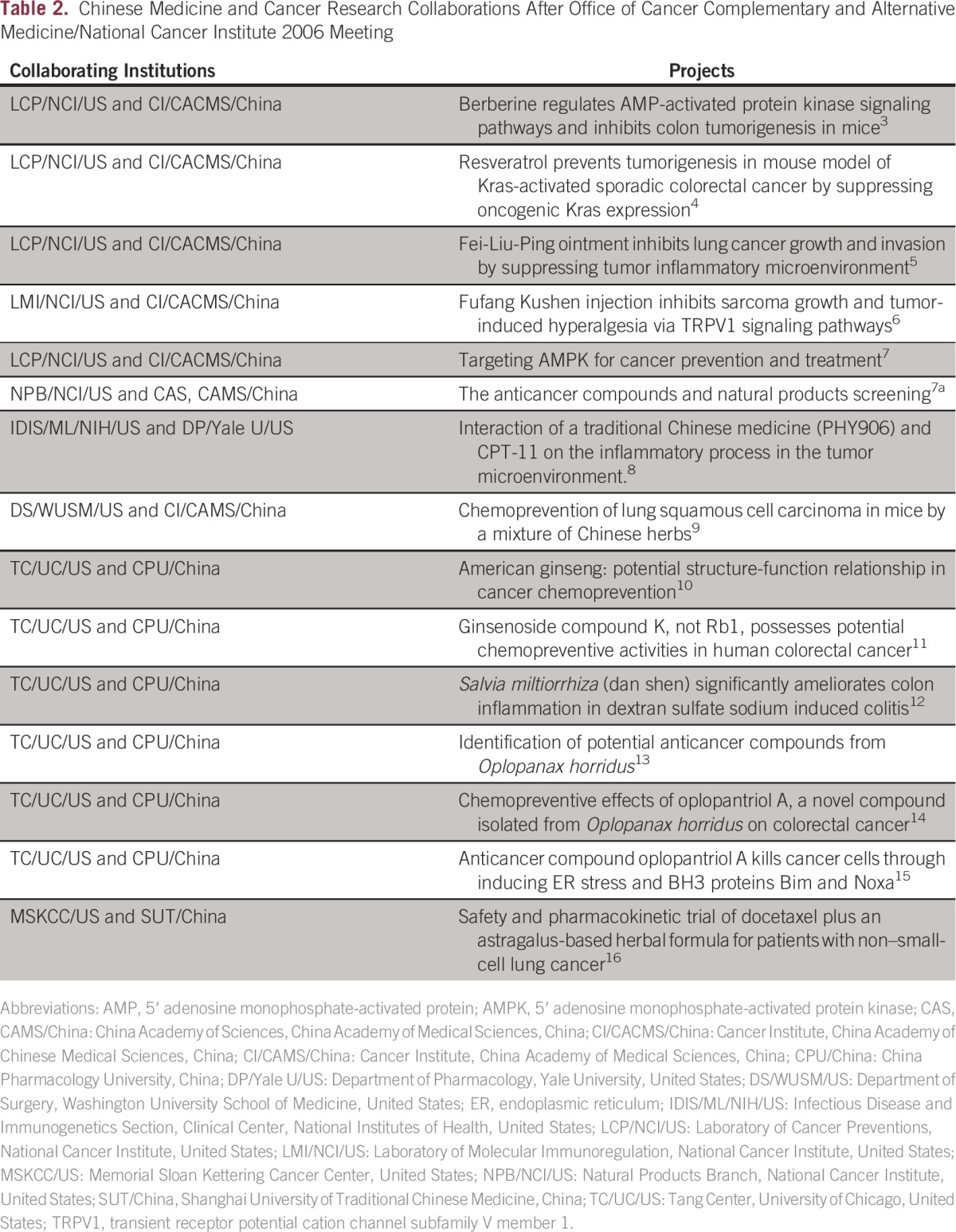
Chinese Medicine and Cancer Research Collaborations After Office of Cancer Complementary and Alternative Medicine/National Cancer Institute 2006 Meeting

In 2011 and 2013, OCCAM and Cancer Institute, China Academy of Chinese Medical Sciences (CACMS) cohosted two conferences in Beijing, China, on CM and cancer research. These meetings continued to explore the intersection of CM and Western oncology and further established the potential value of more dialogue between scientists and health care practitioners from different countries and backgrounds, the potential synergies that can come from joining together complementary resources, and the potential for international scientific communications and collaborations to aid in the modernization of CM for oncology applications.

In February 2014, the Cancer Institute, CACMS, presented OCCAM with a proposal for an international consortium with the aims of improving scientific communications and collaborations, improving the quality of research on CM for oncology applications, finding potential promising CM drugs, and modernizing the research of CM for cancer. NCI’s OCCAM agreed to work with CACMS to explore the feasibility of establishing an international consortium for CM and cancer.

On November 3, 2014, NCI held a meeting at the US National Institutes of Health’s Bethesda, MD, campus to assess the feasibility of developing an international consortium for Chinese medicine and cancer (ICCMC) and to discuss the potential goals and structures for such an international collaboration. Topics and questions proposed by the organizers included: What are the most promising therapies from CM for cancer treatment and management? What are the mechanisms of action of these promising therapies? How can this information help in optimally combining these therapies with conventional Western biomedical approaches? How can these approaches be standardized (eg, plant sourcing and content analysis) so that they provide consistent effects and are optimized (eg, dose and schedule) to provide maximal benefit? What is necessary for academic centers and research hospitals to work effectively with industry to carefully study the clinical utility of CM herbal therapies in cancer management?

## MEETING PROCEEDINGS

### Bridging CM and Modern Biomedicine

Jie Li, MD, PhD, and Hongsheng Lin, MD, from Cancer Institute, CACMS, highlighted one of the main differences between CM and Western oncology: CM focuses on comprehensively adjusting the internal environment of the body, whereas the general goal of Western cancer treatment is to treat the tumor alone. CM uses a clinical assessment process that differs greatly from modern Western medicine. The CM diagnostic approach defines specific body types or constitutions, such as Chi (or Qi) deficient. What are the molecular pathways associated with specific diagnosis patterns? With modern technologies, there are opportunities to examine the molecular biology underlying CM diagnoses. Researchers could potentially explore the genetic background for those types with sufficient sample sizes.

### Recent Clinical Research of Chinese Botanical Medicines

Stephen Lam, MD, discussed a series of phase I and phase II cancer trials of botanical drugs, coordinated by his group at the British Columbia Cancer Agency and performed in Canada and the United States. The drugs his group has tested included the multi-herb agents ACAPHA (also known as antitumor B or Zeng Sheng-ping Pian),^9,17,18^ Aneustat,^19^ myoinositol,^20,21^ and the green tea extract Polyphenon E. All botanicals used in the presented trials were manufactured under good manufacturing practices, with careful standardization. In general, although the safety profile in these trials was good, as stand-alone agents these botanical drugs to date have shown only modest effects for chemoprevention of cancer.

Jie Li, MD, PhD, and Hongsheng Lin, MD, presented promising data on CM applications for cancer treatment and symptom management.^6,22-29^ In an ongoing randomized trial, an herbal medicinal cream is being studied for the treatment of hand-foot syndrome among patients being treated with the chemotherapeutic agent capecitabine. Early results showed significant improvements in pain and restricted mobility caused by epidermal growth factor receptor inhibitor–related skin toxicity.^30^

### Issues of Trial Design and Monitoring With CM

In China, CM practitioners see a large number of patients, but the treatment efficacy has not been well documented in a standardized way. Meeting participants discussed a need among the CM programs in China for research capacity building and for improving the quality of clinical trials.

An outstanding question is whether using therapies selected based on a patient’s CM diagnosis makes a difference in outcomes.^29^ A clinical trial is currently underway to examine the potential benefit of adjuvant therapy with two herbal formulas (Si Jun Zi decoction and Liu Wei Di Huang decoction) for patients with stage II and III colon cancer, one arm of the study in China and one in Norway.^31^ The trial is testing if the use of a CM herbal formula after radical surgery and conventional chemotherapy/radiotherapy could reduce the rate of recurrence and metastasis and prolong the overall survival time.

Dose finding may also be different for herbal formulas than for other pharmaceuticals. For example, in one trial discussed by participants, the highest dose did not provide the best response.^32^ Also, in CM, practitioners can use different herbs for the same goals—the concept of same disease, different medicine.^33^ One formula may not work for everyone with the same tumor type. CM is a complex intervention system, including herbs, diet, exercise, and other components. Some participants expressed concern that when you reduce the system to its components to simplify the research study design, you have the potential to reduce efficacy.

Nagi Kumar, MD, of the Moffitt Cancer Center at the University of South Florida overviewed the literature and her own research showing that botanicals can influence multiple biochemical cascades leading to inhibition of mutagenesis and proliferation, induction of apoptosis, and suppression of formation and growth of human cancers, with a significantly superior safety profile compared with most agents evaluated to date. Kumar presented a systematic approach to accelerate development of CM and discussed the ABCDEs (Agent, Biomarkers, Cohorts for testing, Design, and End points) of evaluating compounds/herbal mixtures for cancer prevention and treatment. Factors to consider when evaluating CM/herbal mixture include ADME (absorption, distribution, metabolism, and excretion) properties and profiles; toxicity; risk/benefit ratio; dose, route, schedule, and half-life; whole compounds or mixtures versus single agents; and standardization and quality control. Chemoprevention biomarkers for a trial should be identified early, clinically relevant, and modulated by a given therapy; they should reliably estimate the end point of interest and have accuracy in predicting a specific cancer.^34-36^

Lizhu Lin, MD, of the First Affiliated Hospital of Guangzhou University of Chinese Medicine, highlighted current issues with published clinical trials of CM for cancer.^37-41^ In a search of ClinicalTrials.gov^42^ and Chinese Clinical Trial Register,^43^ 42 registered trials with published results between 2008 and 2014 were identified. Most of these reports failed to properly document the CM diagnosis according to disease and syndrome differentiation. Only four (9.5%) of the study protocols mentioned syndrome classification and a description of the CM diagnosis. Only two (4.8%) mentioned a blinded design, no study included sample size calculation, and no study performed an intention-to-treat analysis. Measures to ensure patient compliance and quality control of the study drug were inadequate. For clinical trials of CM on cancer in China, Lin indicated that the following are needed: integration of national resources of clinical research centers to build the capacity of multiagency cooperation and to use big data to explore the efficacy of CM on cancer,^41^ identification of the most important clinical opportunities for the use of CM for treatment of common cancers, and selection of the most appropriate therapeutic outcome measures. Measures of quality of life may be particularly important in such trials. On the topic of improving the standards of randomized controlled trials for CM, several participants stressed the importance of extending the CONSORT statement^44^ to herbal medicine trials.

### Quality Control and Standardization of Chinese Botanical Medicines

In a panel session, participants discussed how ensuring the quality of CM products and the consistency between batches is a complex issue, because of regional and seasonal variations in the chemical composition of many herbs. Large companies with steady cash flow are in a position to develop high-quality study agents, and the question was posed whether large Chinese companies would want to partner with investigators in the United States and elsewhere to research these products. Such companies have the financial resources but may not be familiar with international regulatory requirements. A consortium could serve as a conduit for these types of research collaborations to study herbal products from the CM pharmacopeia.

### US Regulatory Issues for Botanical Medicines

Jinhui Dou, MD, a pharmacologist from Botanical Review Team, Center for Drug Evaluation and Research, of the US Food and Drug Administration (FDA), presented on further development of herbal medicines as FDA-approved new botanical drug products through investigational new drug application and new drug application (NDA) processes.^45,46^ For developing a botanical drug from medicinal plants, purification of the mixture into single molecules is not required (but partial purification is encouraged), and identification of active constituents is not essential. Experiences from prior human use may substitute for some of the animal toxicology studies to support the safety of early-phase clinical trials. With extensive human experience to be reviewed as adequate to support safety, nonclinical evaluations may be reduced or delayed until when the phase III trials are planned. With a well-documented history of prior human use, most of the botanicals can advance directly to single-dose or multiple-dose phase II trials. Adequate and well-controlled trials are necessary for marketing CM and other herbal products as botanical drugs in the United States. When late-phase clinical studies are being considered to demonstrate the clinical safety and efficacy of the botanical products for an NDA, the FDA requires the same level of clinical efficacy/safety requirements as nonbotanical drugs for new drug market approvals. Because botanicals are complex mixtures, multiple-dose and multiple-batch phase III clinical trials can be useful tools to demonstrate consistency of the botanical products, when practical. Dou highlighted two case studies of botanical products that received FDA approval as NDAs in the past decade. The first was the topical agent Veregen from MediGene (Planegg/Martinsried, Germany),^47^ for treating genital/perianal warts (investigational new drug in 1996). The active ingredient is a partially purified green tea extract, mainly catechins. The NDA for Veregen was approved in 2006. The second example was the oral agent Fulyzaq (crofelemer)^48^ for HIV-related diarrhea. The botanical raw material for producing the drug substance (crofelemer) is crude plant latex from a South American plant species, *Croton lechleri* (commonly known as dragon’s blood). The NDA sponsor of Fulyzaq was Salix Pharmaceuticals (Raleigh, NC), and it was approved in 2012. Lessons learned from the two botanical NDAs include that botanical raw material controls are required, with adoption of good agriculture and collection practices, and that specifications for drug substance/product are acceptable for chemical batch equivalence, but alone may be inadequate; non–Chemistry Manufacturing and Controls data (eg, bioassays) may be needed to ensure therapeutic consistency.

### Working With Other CM–Related Organizations

Lixing Lao, MD, PhD, Director of the School of Chinese Medicine, University of Hong Kong, introduced an existing consortium for which he serves as the secretary general, which brings together CM and Western medicine researchers and practitioners: The Consortium for Globalization of Chinese Medicine (CGCM),^49^ founded in 2003. It currently has 144 institutional members and 18 industrial affiliate members. The CGCM has also spawned an offshoot, the Good Practice in CM Research Association, launched in April 2012 in the Netherlands. The meeting participants believed that the CGCM has a good system for recruiting academic institutions and industry and that lessons could be learned from the CGCM in how to establish this new consortium in a stable way. The CGCM focuses on all aspects of CM; therefore, collaborations focused purely on CM and cancer treatment have the potential to add value to both CGCM and the emergent ICCMC.

### Consortium Funding Mechanisms and Structures

Roy Wu, PhD, Chief of the Clinical Grants and Contracts Branch of NCI’s Division of Cancer Treatment and Diagnosis, presented potential funding models for a consortium. He stressed that the ideal consortium structure will depend on what participants want to accomplish, but a secondary issue will be the type of funding pursued, because certain types of government grants require certain organizational structures. Among the possible funding mechanisms for a consortium is the NIH Cooperative Agreement program (U01). This mechanism has been used to support various types of clinical trials networks, as exemplified by the existing Cancer Immunotherapy Network,^50^ Blood and Marrow Transplant Clinical Trials Network,^51^ and Experimental Therapeutics Clinical Trial Network.^52^ The cooperative agreement is similar to a grant mechanism, but it requires participation by NIH staff. Additional outside funding is allowed, as long as there is no overlap in how the funding is used. A center of excellence was proposed as another potential organizational structure. At the NIH these centers are often supported by P50 grants.^53^ The potential also exists to organize a consortium with multiple networks of activities and different levels of funding or even involve some industry partners, so the consortium would not be defined or restricted by its funding. Regarding the work of the consortium, participants suggested that a registry project may be a good start for establishing a structure for collaboration; this is how the Academic Consortium for Integrative Medicine and Health^54^ began.

### Future Directions

The participants gave some possible early project suggestions, including developing exemplary guidelines for CM oncology clinical trials, developing standard treatment protocols and evaluation procedures (like National Comprehensive Cancer Network guidelines), and creating educational exchanges to find common ground and develop research capacity. The sentiment that it is time to solidly act, not just talk, was expressed by many participants. Pick a smaller, straightforward project and use that to establish collaborative working relationships and figure out what may and may not work for a consortium. Levels of complexity can be added as the collaborations grow and mature. The first meeting of the consortium was planned and held on October 16 to 18, 2015 in Dalian, China.

## References

[1] Lu H, Yang Y, Gad E (2011). Polysaccharide krestin is a novel TLR2 agonist that mediates inhibition of tumor growth via stimulation of CD8 T cells and NK cells. Clin Cancer Res.

[2] Office of Cancer Complementary and Alternative Medicine: OCCAM conferences. http://cam.cancer.gov/news_and_events/conferences.htm

[3] Li W, Hua B, Saud SM (2015). Berberine regulates AMP-activated protein kinase signaling pathways and inhibits colon tumorigenesis in mice. Mol Carcinog.

[4] Saud SM, Li W, Morris NL (2014). Resveratrol prevents tumorigenesis in mouse model of Kras activated sporadic colorectal cancer by suppressing oncogenic Kras expression. Carcinogenesis.

[5] Li W, Chen C, Saud SM (2014). Fei-Liu-Ping ointment inhibits lung cancer growth and invasion by suppressing tumor inflammatory microenvironment. BMC Complement Altern Med.

[6] Zhao Z, Fan H, Higgins T (2014). Fufang Kushen injection inhibits sarcoma growth and tumor-induced hyperalgesia via TRPV1 signaling pathways. Cancer Lett.

[7] Li W, Saud SM, Young MR (2015). Targeting AMPK for cancer prevention and treatment. Oncotarget.

[7a] National Cancer Institute: Rare plant compounds from China undergo screening at NCI. http://cam.cancer.gov/docs/newsletter/cam-news-spring-2009.pdf

[8] Wang E, Bussom S, Chen J (2011). Interaction of a traditional Chinese Medicine (PHY906) and CPT-11 on the inflammatory process in the tumor microenvironment. BMC Med Genomics.

[9] Wang Y, Zhang Z, Garbow JR (2009). Chemoprevention of lung squamous cell carcinoma in mice by a mixture of Chinese herbs. Cancer Prev Res (Phila).

[10] Qi L-W, Wang C-Z, Yuan C-S (2010). American ginseng: Potential structure-function relationship in cancer chemoprevention. Biochem Pharmacol.

[11] Wang CZ, Du GJ, Zhang Z (2012). Ginsenoside compound K, not Rb1, possesses potential chemopreventive activities in human colorectal cancer. Int J Oncol.

[12] Wen X-D, Wang C-Z, Yu C (2013). Salvia miltiorrhiza (dan shen) significantly ameliorates colon inflammation in dextran sulfate sodium induced colitis. Am J Chin Med.

[13] Wang C-Z, Zhang Z, Huang WH (2013). Identification of potential anticancer compounds from Oplopanax horridus. Phytomedicine.

[14] Zhang Z, Yu C, Zhang CF (2014). Chemopreventive effects of oplopantriol A, a novel compound isolated from Oplopanax horridus, on colorectal cancer. Nutrients.

[15] Jin HR, Liao Y, Li X (2014). Anticancer compound oplopantriol A kills cancer cells through inducing ER stress and BH3 proteins Bim and Noxa. Cell Death Dis.

[16] Cassileth BR, Rizvi N, Deng G (2009). Safety and pharmacokinetic trial of docetaxel plus an Astragalus-based herbal formula for non-small cell lung cancer patients. Cancer Chemother Pharmacol.

[17] Zhang Z, Wang Y, Yao R (2004). Cancer chemopreventive activity of a mixture of Chinese herbs (antitumor B) in mouse lung tumor models. Oncogene.

[18] Lin P, Zhang J, Rong Z (1990). Studies on medicamentous inhibitory therapy for esophageal precancerous lesions--3- and 5-year inhibitory effects of antitumor-B, retinamide and riboflavin. Proc Chin Acad Med Sci Peking Union Med Coll.

[19] Qu S, Wang K, Xue H (2014). Enhanced anticancer activity of a combination of docetaxel and Aneustat (OMN54) in a patient-derived, advanced prostate cancer tissue xenograft model. Mol Oncol.

[20] Lam S, McWilliams A, LeRiche J (2006). A phase I study of myo-inositol for lung cancer chemoprevention. Cancer Epidemiol Biomarkers Prev.

[21] Gustafson AM, Soldi R, Anderlind C, et al: Airway PI3K pathway activation is an early and reversible event in lung cancer development. Sci Transl Med 2:26ra25, 2010 10.1126/scitranslmed.3000251 PMC3694402 20375364

[22] Li J, Sun G-Z, Lin H-S (2008). The herb medicine formula “Yang Wei Kang Liu” improves the survival of late stage gastric cancer patients and induces the apoptosis of human gastric cancer cell line through Fas/Fas ligand and Bax/Bcl-2 pathways. Int Immunopharmacol.

[23] Li J, Lin H, Wang X (2009). Molecular mechanisms of traditional chinese medicine re-sculpture effect on the process of tumor immunoediting. Wood Sci Technol.

[24] Lin H, Liu J, Zhang Y (2011). Developments in cancer prevention and treatment using traditional Chinese medicine. Front Med.

[25] Zhang Y, Piao B, Zhang Y (2011 (suppl 1)). Oxymatrine diminishes the side population and inhibits the expression of β-catenin in MCF-7 breast cancer cells. Med Oncol.

[26] Xu W, Lin H, Zhang Y (2011). Compound Kushen injection suppresses human breast cancer stem-like cells by down-regulating the canonical Wnt/β-catenin pathway. J Exp Clin Cancer Res.

[27] Li J, Lin HS, Hou W (2011). Development and current status of National Cancer Center for Chinese medicine. Chin J Integr Med.

[28] Li J, Lin HS (2011). Integrative medicine: A characteristic China model for cancer treatment. Chin J Integr Med.

[29] Li J, Li L, Liu R (2012). Establishing Chinese medicine characteristic tumor response evaluation system is the key to promote internationalization of Chinese medicine oncology. Chin J Integr Med.

[30] Lin H, Li J. Effect of Chinese herbal medicine JDXZO on epidermal growth factor receptor inhibitor (EGFRIs)-related skin toxicity. J Clin Oncol 29, 2011 (suppl; abstr TPS243)

[31] US National Institutes of Health: Comprehensive therapy to relieving the risk of recurrence and metastasis for colorectal cancer. https://clinicaltrials.gov/ct2/show/NCT00689364?term=Yang+Yufei&rank=1

[32] Hong DS, Henary H, Falchook GS (2014). First-in-human study of pbi-05204, an oleander-derived inhibitor of akt, fgf-2, nf-κΒ and p70s6k, in patients with advanced solid tumors. Invest New Drugs.

[33] Liu Y, Fang T, Vian K, et al: The Essential Book of Traditional Chinese Medicine: Clinical Practice, Theory. Columbia University Press, 1988

[34] Meyskens FL, Curt GA, Brenner DE (2011). Regulatory approval of cancer risk-reducing (chemopreventive) drugs: Moving what we have learned into the clinic. Cancer Prev Res (Phila).

[35] Connors SK, Chornokur G, Kumar NB (2012). New insights into the mechanisms of green tea catechins in the chemoprevention of prostate cancer. Nutr Cancer.

[36] Kumar N, Crocker T, Smith T (2012). Challenges and potential solutions to meeting accrual goals in a phase II chemoprevention trial for prostate cancer. Contemp Clin Trials.

[37] Wang S, Lin L, Zhou J (2011). Effects of yiqi chutan tang on the proteome in Lewis lung cancer in mice. Asian Pac J Cancer Prev.

[38] Lin LZ, Li P, Chen HR (2013). Sunitinib malate as first-line treatment for an advanced, poorly differentiated pancreatic neuroendocrine tumor. Future Oncol.

[39] Zhai L, Zhao Y, Lin L (2012). Non-Hodgkinʼs lymphoma involving the ileocecal region: A single-institution analysis of 46 cases in a Chinese population. J Clin Gastroenterol.

[40] Wang SM, Lin LZ, Zhou DH (2015). Expression of prolyl 4-hydroxylase beta-polypeptide in non-small cell lung cancer treated with Chinese medicines. Chin J Integr Med.

[41] Chen CM, Lin LZ, Zhang EX (2014). Standardized treatment of Chinese medicine decoction for cancer pain patients with opioid-induced constipation: A multi-center prospective randomized controlled study. Chin J Integr Med.

[42] US National Institutes of Health: ClinicalTrials.gov. https://www.clinicaltrials.gov/

[43] Chinese Clinical Trial Registry. http://www.chictr.org.cn/

[44] Begg C, Cho M, Eastwood S (1996). Improving the quality of reporting of randomized controlled trials: The CONSORT statement. JAMA.

[45] Chen ST, Dou J, Temple R (2008). New therapies from old medicines. Nat Biotechnol.

[46] Lee SL, Dou J, Agarwal R, et al: Evolution of traditional medicines to botanical drugs. Science 347:S32-S34, 2015 (suppl 6219)

[47] (2008). Med Lett Drugs Ther.

[48] MacArthur RD, Hawkins TN, Brown SJ (2013). Efficacy and safety of crofelemer for noninfectious diarrhea in HIV-seropositive individuals (ADVENT trial): A randomized, double-blind, placebo-controlled, two-stage study. HIV Clin Trials.

[49] Consortium for Globalization of Chinese Medicine. http://www.tcmedicine.org/en/default.asp

[50] Cancer Immunotherapy Trials Network. http://citninfo.org/

[51] Blood and Marrow Transplant Clinical Trials Network. http://www.bmtctn.net/10.1016/j.bbmt.2016.07.003 PMC5027144 27418009

[52] National Cancer Institute: NCI Experimental Therapeutics Clinical Trials Network (ETCTN). http://ctep.cancer.gov/initiativesPrograms/etctn.htm

[53] National Institutes of Health: Grants & Funding: Activity codes search results. http://grants.nih.gov/grants/funding/ac_search_results.htm?text_curr=p50&Search_Type=Activity

[54] Academic Consortium for Integrative Medicine and Health. http://www.imconsortium.org/

